# Necroptosis-related lncRNA signature predicts prognosis and immune response for cervical squamous cell carcinoma and endocervical adenocarcinomas

**DOI:** 10.1038/s41598-022-20858-5

**Published:** 2022-09-29

**Authors:** Zhiheng Lin, Jiani Zou, Xiaohui Sui, Shujuan Yao, Lidong Lin, Jiuling Wang, Junde Zhao

**Affiliations:** 1 Shandong University of Traditional Chinese Medicine, Jinan, 250014 Shandong China; 2 Affiliated Hospital of Shandong University of Traditional Chinese Medicine, Jinan, 250014 Shandong China; 3 Office of Medical Insurance Management, Qilu Hospital of Shandong University, Jinan, 250012 China

**Keywords:** Cancer, Computational biology and bioinformatics, Genetics, Immunology, Molecular biology, Biomarkers

## Abstract

Necroptosis, a programmed form of necrotic cell death, plays critical regulatory roles in the progression and metastatic spread of cancers such as cervical squamous cell carcinoma and endocervical adenocarcinoma (CESC). However, there are few articles systematically analyzing the necroptosis-related long non-coding RNAs (NRlncRNAs) correlated with CESC patients. Both RNA-sequencing and clinical data of CESC patients are downloaded from TCGA database in this study. Pearson correlation analysis, least absolute shrinkage, operator algorithm selection and Cox regression model are employed to screen and create a risk score model of eleven-NRlncRNAs (MIR100HG, LINC00996, SNHG30, LINC02688, HCG15, TUBA3FP, MIAT, DBH-AS1, ERICH6-AS1SCAT1, LINC01702) prognostic. Thereafter, a series of tests are carried out in sequence to evaluate the model for independent prognostic value. Gene set enrichment analytic paper, Gene Ontology analysis, Kyoto Encyclopedia of Genes and Genomes pathway enrichment analytic paper make it clear that immune-related signaling pathways are very rich in the high-risk subgroup. Additionally, the prognostic risk score model is correlated to immune cell infiltration, potential immune checkpoint, immune function, immune micro-environmental and m6A-related gene. Mutation frequency in mutated genes and survival probability trend are higher in the low-risk subgroup in most of test cases when compared to the high-risk subgroup. This study constructs a renewed prognostic model of eleven-NRlncRNAs, which may make some contribution to accurately predicting the prognosis and the immune response from CESC patients, and improve the recognition of CESC patients and optimize customized treatment regimens to some extent.

## Introduction

Cervical cancer is the second most prevalent gynecological cancer^1^, one of leading causes of female death due to cancer in the world^2^. Cervical cancer is globally developing. More than 270,000 women worldwide die from cervical cancer each year^3^, especially in developing countries^4,5^. In the case of cervical cancer, the cervical squamous cell carcinoma and endocervical adenocarcinomas (CESC) accounts for about 10 to 15% of all tumor-related, female deaths^6^. The prognosis remains weak for patients at the terminal stage of CESC and most of CESC patients may have already gone into the middle or terminal stages when they get diagnosed initially, despite that the technology for diagnosis and treatment of CESC has developed a lot over the past 30 years. It is greatly significant to develop a novel and validated biomarker for CESC prognosis to guide clinical experience. With the deepening study of the cell death mechanisms, more and more novel cell-death modalities have been discovered and named. Necroptosis is a form of regulatory necrotic cell death adjusted by receptor-interacting protein [RIP] kinase 1(RIPK1), RIPK3 and activated by mixed lineage kinase domain-like pseudo kinase (MLKL), which was discovered and named in 2005 Degterev et al.^7^. Current studies have pointed out that necroptosis had the double roles in promoting and suppressing tumor and that a novel strategy to treat cancers might be – targeting necroptosis^8^. Park et al. reported that RIPK1 overexpression inhibited p53 pathway through the activation of NF-kappaB and that it had to do with a worse prognosis in glioblastoma^9^. A recent study showed that RIPK3 was a critical regulator in the malignant transformation of hepatocellular carcinoma (HCC) and that to regulate necroptosis may improve the chemosensitivity of HCC^10^. And by the same token, necroptosis has an important effect on cervical cancers. More tantalizingly, MLKL is a prognostic biomarker for the pancreatic cancer and the increased expression of MLKL may improve the overall survival rate^11^. There was a study which indicated that the expression status of RIPK3 acted as a critically influential factor for the release of IL-1α and the outcome of PolyIC-based immunotherapeutic approaches in cervical cancer cells^12^. These studies all showed that the key necroptotic factors which were mediated by escaping from necroptosis, presented one of the relevant molecular mechanisms of CESC progression and metastasis. There may be valuable implications for the treatment and prognostication of CESC patients in the future to induce necroptosis. Nevertheless, there is nearly no researches on the association between CESC and necroptosis in a wide and deep manner at present and the academia doesn’t figure out the clear role of necroptosis in the prognosis of CESC patients. Therefore, the team of this study needs to find out more novel necroptosis-related biomarkers for CESC patients to identify better treatments and predict prognostic effects in a better way.

Long non-coding RNAs (lncRNAs) are a subset of non-coding RNAs produced by the transcription of RNA polymerase II, which is 200-nucleotide longer in length^13^ and lacks the function of protein-coding^14^. A variety of lncRNAs expressions are abnormal in the tissues of cervical cancer, which is pointed out in some studies, and the expression level is significantly correlated with the invasion, apoptosis, proliferation and metastasis of cervical cancer cells in addition to the proliferation and metastasis of cervical cancer^15^. lncRNAs may help cervical cancer cells in combatting radiotherapy and chemotherapy, thus to promote the growth of cancer^16^. However, few systematically analytical papers focused on NRlncRNAs correlated to the prognosis of cervical cancer.

It is proven that high-throughput sequencing technology and bioinformatics approaches help explore valuable disease-specific biomarkers. This study investigated the molecular and signaling pathways recognizing NRlncRNAs so that the pavement can be provided for the research and development of a renewed risk-score model to predict the prognosis and select the immunotherapies of CESC patients. The first NRlncRNAs signature model with CESC was constructed, which may provide a new perspective to discover more accurate treatments for CESC patients.

## Materials and methods

### Raw data acquisition

Both mRNA and lncRNA sequencing data were selected from 303 CESC cases and 3 normal cervical tissue samples in The Cancer Genome Atlas (TCGA) database (https://portal.gdc.cancer.gov) and R (R 4.1.2, https://www.r-project.org/) was used to extract and standardize the data. A total of 104 necroptosis-related genes (NRGs) were retrieved from previous researches provided in the Appendix Table S1. The Pearson correlation coefficients were also calculated to evaluate the connection between lncRNAs and NRGs to identify lncRNAs related to NRGs. The absolute value of Pearson correlation coefficients greater than 0.4 (|R|> 0.4) and the *p* value less than 0.001 (*p* < 0.001) were used to filter the NRGs-related lncRNAs (NRGlncRNAs). Both LncRNA expression profile and relevant clinical data of CESC tissue samples originated from the GEO database (http://www.ncbi.nlm.nih.gov/geo). The TCGA samples, as validation datasets, were randomly divided into two equal numbers of groups and they were named Test1 and Test2, respectively. Since the TCGA database is publicly accessible, this study is exempted from the approval of the Ethics Committee. R is a software environment for statistical computing and graphics, which can be used for free. It compiles and runs on a wide variety of UNIX platforms, Windows and MacOS. R version 4.1.2; link: https://www.r-project.org/.

### Construction and validation of a prognostic necroptosis-related lncrnas signature

Univariate Cox regression analysis is used to perform the screening of overall survival (OS) and identify the OS-related prognostic lncRNAs in the extracted genes (*p* < 0.05). Fifty-six genes associated with survival were selected for the further analysis. The least absolute shrinkage and selection operator (LASSO) are used to construct the Cox regression model using R software ‘glmnet’ package in order to precisely secure the range of the candidate genes and construct the prognostic model^17,18^. The risk gene was obtained through multivariate Cox regression analysis, while the risk score model was constructed to predict the survival time and the risk score coefficient (coefi) was included in the gene output of the model. According to the median risk score, these cases are categorized into two risk subgroups. The following is the calculation formula of the risk score model (X: coefficients, Y: gene expression level).

$$ {\text{Risk}}\;{\text{Score}} = \mathop \sum \limits_{{\text{i}}}^{{\text{n}}} {\text{Xi}} \times {\text{Yi}} $$

### Predictive value of the LncRNA model

The principal component analysis (PCA) and t-SNE analysis were carried out to explore the data distribution for two risk subgroups and visualize the two groups using the “Rtsne” and “ggplot2” packages in R. The OS of the two risk subgroups were compared in the risk scores using the “survival” package. The “Time ROC” and “Clinical ROC” package were further used to evaluate the prediction accuracy and visualize the distribution of risk scores across clinical data. Univariate and multivariate Cox regression analyses were performed to evaluate the association between variables, while risk values and prognosis were used to test whether the risk model acts as an independent prognostic index in the treatment of CESC or not. A nomogram was used to predict the prognosis of CESC patients using the “rms” package. Calibration curves were plotted to compare and verify the survival probability rate of 1, 3 and 5-year nomogram prediction.

### Construction of the mRNA-lncRNA co-expression network

The mRNA-lncRNA co-expression network was structured and visualized using Cytoscape software (software information is version 3.7.2 at http://www.cytoscape.org/) to additionally figure out the potential relationship and interaction between NRlncRNAs and their corresponding mRNAs.

### Gene set enrichment analysis

Gene set enrichment analysis (GSEA; http://www.broadinstitute.org/gsea) was conducted to explore the potential functional pathways or molecular mechanisms that contain the differentially-expressed NEGs between the high-risk and low-risk groups^19^. Min and max genomes of 15 and 500 genes were used to screen the NRGs sets.

### Functional enrichment analysis

The Kyoto Encyclopedia of Genes and Genomes (KEGG) pathway analysis and the Gene Ontology (GO) analysis were conducted to identify the molecular functions (MF), cellular components (CC), biological process (BP), and NRlncRNAs-related signaling pathways.

### Immune and M6A correlation analysis

A single-sample gene set enrichment analysis (ssGSEA) was performed to contrast the immune cell infiltration performance between the high-risk and low-risk groups and to discover the immune function^20,21^. Potential immune checkpoints were used to find out the association between immune-related genes and risk signatures. The immune microenvironment scores including stromal and immune scores were calculated using the “ESTIMATE” package, and the 'reshape2' and 'ggpubr' packages were used to visualized^22^. Spearman correlation analysis was conducted to investigate the connection between risk characteristics and M6A-related genes.

### Grouping and survival analysis of TMB

The waterfall function within the "maftools" package was used to calculate the mutation frequency for every CESC sample and display the top 20 mutated genes in two high-risk and low-risk subgroups on a case-by-case basis. CESC samples were categorized into the high and low TMB groups based on the median value of tumor mutation burden (TMB). Kaplan–Meier analysis was carried out to compare the survival difference between the two TMB subgroups and the high-risk group and low-risk group.

## Results

### Construction of the prognostic model based on necroptosis-related lncRNAs for CESC

**Figure 1:**
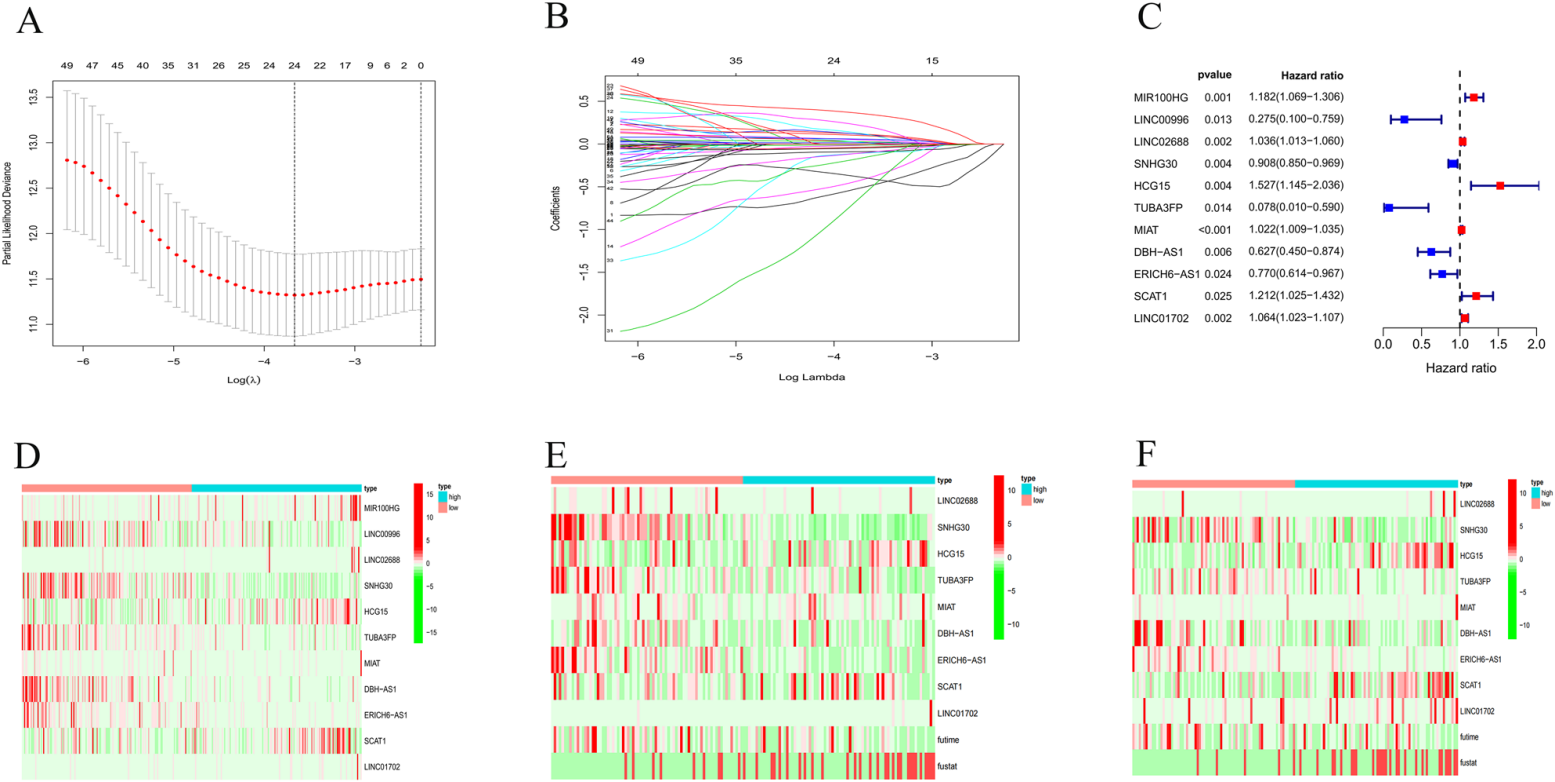
Identification of prognostic necroptosis-related lncRNAs in CESC patients. (**A**, **B**) Lasso Cox regression analysis showed that 24 of the 56 necroptosis-related lncRNAs were good candidates for construction prognostic model. (**C**–**F**) Forest plots and a heatmap (green: low expression level, red: high expression level) with two sets of validation datasets of the 11 necroptosis-related lncRNAs.

Univariate Cox regression analysis on OS was preliminarily carried out to identify 56 survival-related prognostic lncRNAs for the following analysis. Highly-correlated genes were screened out using the LASSO Cox regression model in order to prevent overfitting. The results showed that 24 out of 56 NRlncRNAs were beneficial to CESC prognostic model (Fig. 1A,B). And the Multivariate Cox regression analysis was adopted, and 11 NRlncRNAs including MIR100HG, LINC00996, LINC02688, SNHG30, HCG15, TUBA3FP, MIAT, DBH-AS1, ERICH6-AS1, SCAT1, LINC01702, were obtained eventually to develop an OS-related prognostic model (Fig. 1C–F).

**Figures 2:**
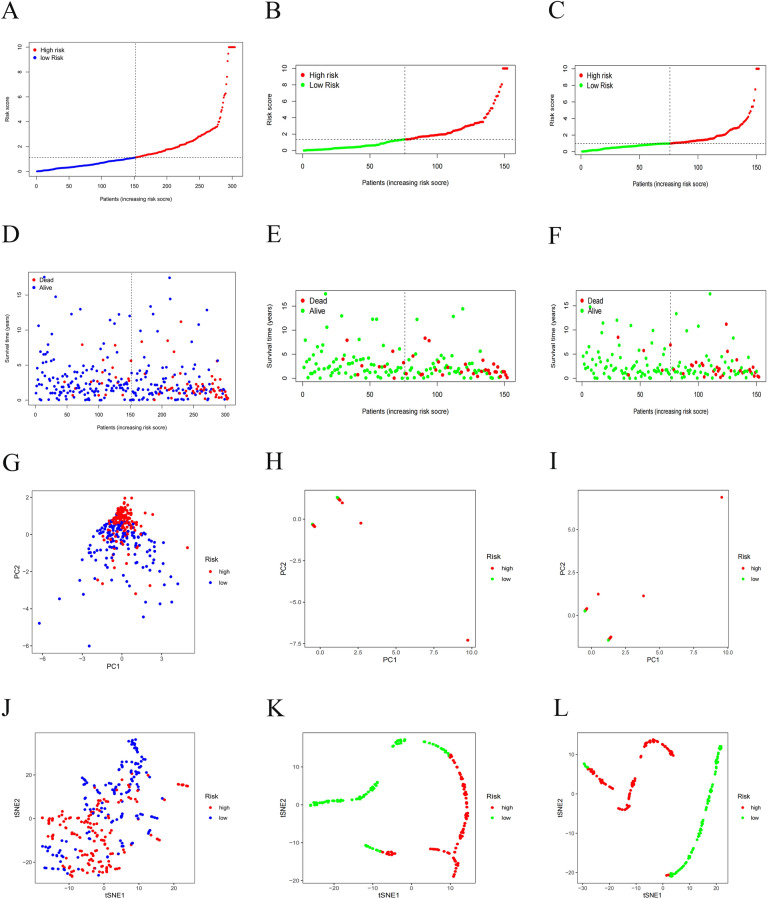
Construction of the prognostic model based on necroptosis-related lncRNAs for CESC. (**A**–**C**) Risk score distribution with two sets of validation datasets, (**D**–**F**) survival status with two sets of validation datasets, (**G**–**I**) PCA plot, (**J**–**L**) t-SNE analysis with two sets of validation datasets of high and low-risk patients.

The median risk-score was used as a cut-off value to category CESC patients into low-risk and high-risk groups (Fig. 2A,D). PCA and t-SNE analyses confirmed that patients with CESC in the two risk subgroups were clearly distinguishable (Fig. 2G,J). At the same time, two sets of validation datasets were constructed to illustrate the generalization capacity of the models and the effect in other cohorts (Fig. 2B,C,E,F,H,I,K–L).

### Associations between clinical characteristics and risk scores in CESC

**Figure 3:**
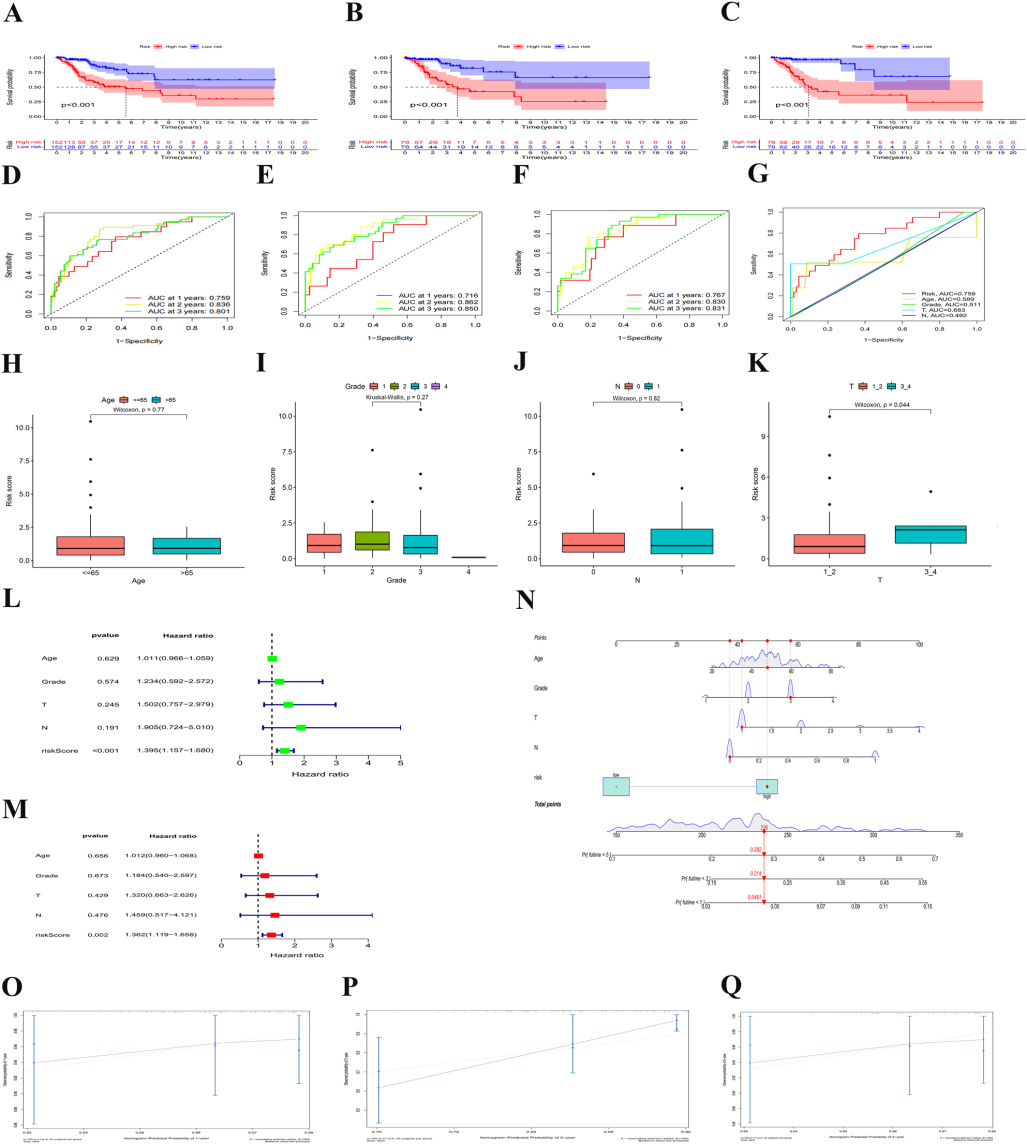
Associations between risk signature and clinicopathological factors. (**A**–**C**) Survival curve of CESC patients. (**D**–**F**) TimeROC curves with two sets of validation datasetsand and (**G**) Clinical ROC curves to forecast overall survival of patients. Visualization of risk scores in clinical data (**H**–**K**). Univariate (**L**) and multivariate Cox (**M**) regression of clinicopathological features in TCGE-CESC cohort. (**N**) Nomogram constructed to predit OS rates at 1, 3 and 5 years. (**P**–**Q**) The nomagram calibration curves were applied for investigation the deviation in nomagram-predicated and actual 1-, 3-, and 5-years survival probabilities.

As shown in Fig. 3A–C, CESC patients in the low-risk group had a higher OS than that in the high-risk group. Through the time receiver operating characteristic (ROC) curve analysis, it was validated that the risk signature had moderate predictive accuracy at 1 (ROC = 0.759), 2 (ROC = 0.836) and 3 (ROC = 0.801) years (Fig. 3D). The remaining two datasets also supported the conclusion: The model had moderate prediction accuracy (Fig. 3E,F). At the same time, the Clinical ROC curve analysis proved that the signature had higher accuracy than other common clinicopathologic parameters (Fig. 3G). The prior data confirmed that the risk signature was a specific and sensitive indicator to predict the OS of CESC. The distribution of risk scores across clinical data was visualized (Fig. 3H–K).

The risk score was highly related to the OS of CESC patients according to multivariate and univariate Cox regression models, indicating that the risk score was an independent prognostic factor and that the above mentioned NRlncRNAs had independent prognostic benefits for CESC patients (Fig. 3L,M). Eventually, a nomogram was constructed to more accurately predict the survival outcome of CESC patients using the risk signature (Fig. 3N). Calibration curves were also used to verify the model's prediction accuracy (Fig. 3O–Q). The data showed that the 1, 3 and 5-year survival rate was in the high consistency with the actual survival rate according to the prediction of the constructed nomogram, which suggested a high prediction value.

### Expression of the mRNA–lncRNA co-expression network

**Figure 4:**
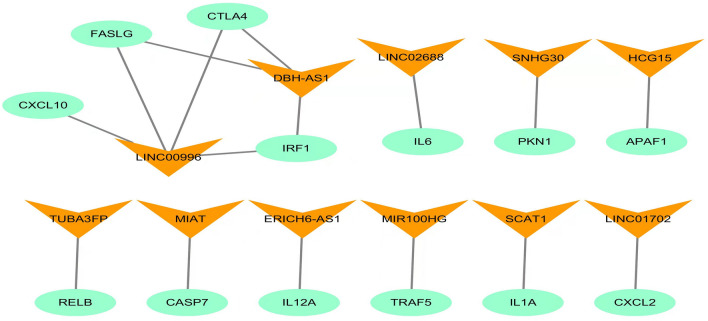
Construction of the necroptosis-related lncRNA-mRNA co-expression network (**A**) Diagram of the necroptosis-related lncRNA-mRNA network.

To investigate the potential effect of the 11 NRlncRNAs in CESC, Cytoscape was employed to construct the mRNA–lncRNA co-expression network. The lncRNA-mRNA co-expression network was involved in 16 mRNA–lncRNA pairs (Fig. 4). Of these, LncRNA LINC00996 was co-expressed with 4 NRGs (CXCL10, FASLG, CTLA4, IRF1), and LncRNA DBH − AS1 had the co-expression connection with 3 NRGs (FASLG, CTLA4, IRF1).

### Discovery of immunoregulatory pathways by GSEA

**Figure 5:**
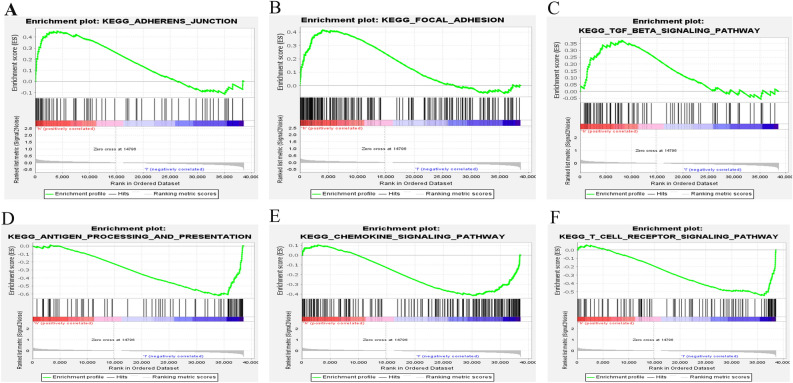
Gene set enrichment analysis (GSEA) of high-risk group and low-risk group based on the necroptosis-related lncRNAs prognostic signature. (**A**–**C**) GSEA results show significant enrichment of adherens junction, focal adhesion, and TGF beta signaling pathway in the high-risk CESC patients. (**D**–**F**) GSEA results show significant enrichment of antigen processing and presentation, Chemokine signaling pathway and T cell receptor signaling pathway in the low-risk CESC patients.

The GSEA was used to figure out the differences in the expressed NRGs between the two risk categories in the TCGA cohort to investigate the differential biological functions and the underlying signaling pathways associated with the NRlncRNAs. The findings revealed that “adherens junction”, “focal adhesion”, and “TGF beta signaling pathway” were all particularly active (Fig. 5A–C) in high-risk CESC patients. The results indicated that high risk was strongly associated with the occurrence, growth and metastasis of tumor. It was found that anti-cancer immunomodulatory pathways were distinctly up-regulated in the low-risk CESC patients such as antigen processing and presentation, chemokine signaling pathway and T cell receptor signaling pathway (Fig. 5D–F).

### Analysis of *GO* terms and *KEGG* pathway enrichment

**Figure 6:**
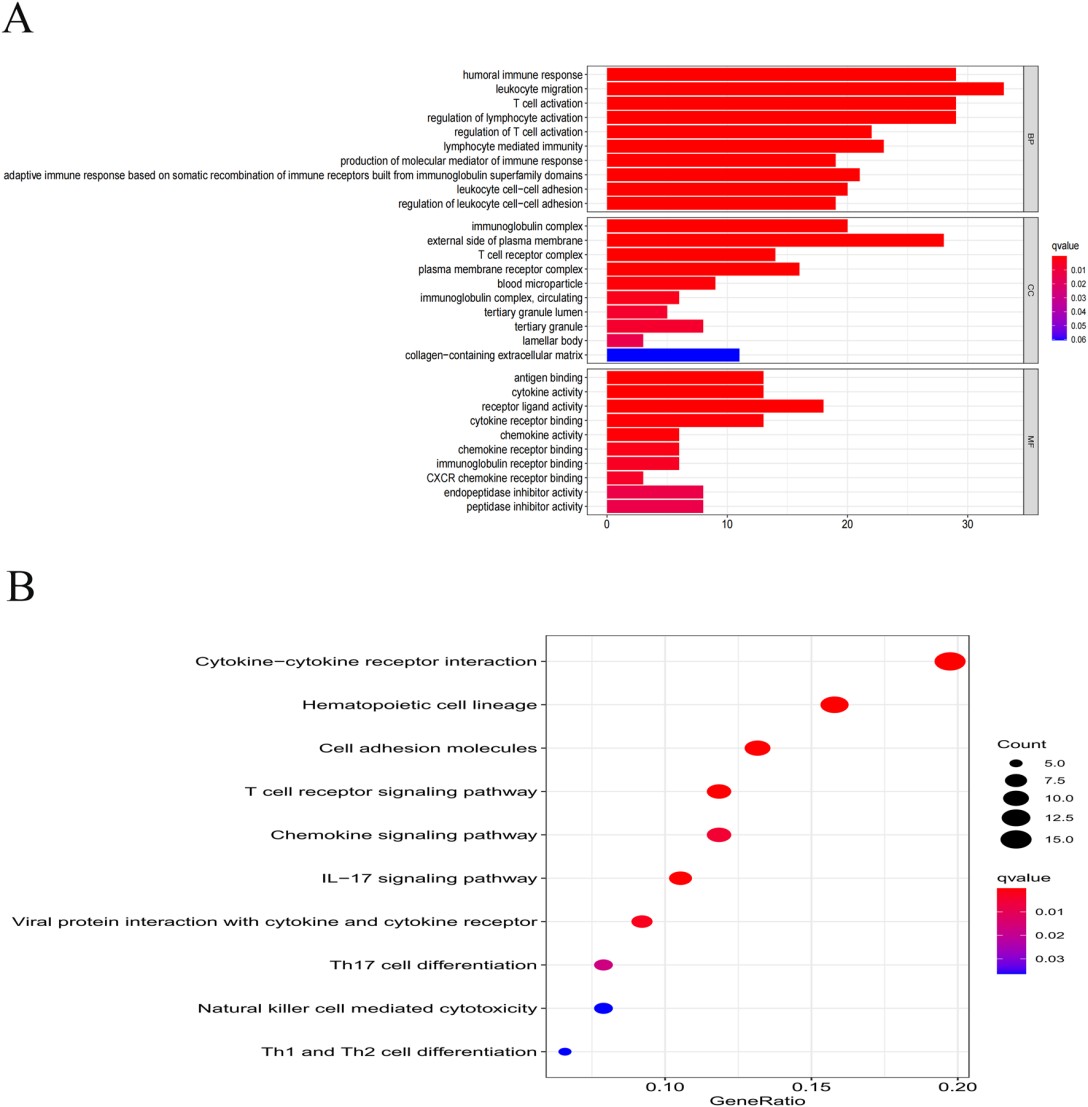
Results of GO and KEGG analyses. (**A**) GO analysis showed differentially expressed genes between high- and low-risk groups were obviously enriched in immune-related biological processed, immune-related cell components. (**B**) KEGG analysis showed differentially expressed genes were enriched in immune-related pathway.

The GO analysis and KEGG pathway enrichment analyses were performed to progressively illuminate significant gene functions of the identified NRlncRNAs in CESC. The finding was that many classifications related to the immune processes showed significant enrichment changes by performing the GO enrichment analysis of differential expression levels of NEGs, especially in immune-related BP such as leukocyte migration, humoral immune response, T cell activation, regulation of lymphocyte activation and CC such as immunoglobulin complex, plasma membrane receptor complex and so on (*p* < 0.05; Fig. 6A). Further stated, KEGG pathway analyses demonstrated that the differentially-expressed genes were conspicuously enriched in cytokine -cytokine receptor interaction, hematopoietic cell lineage, cell adhesion molecules, T cell receptor signaling pathway, chemokine signaling pathway and so on (*p* < 0.05; Fig. 6B).

### Associations with Immunity and M6A-related Genes

**Figure 7:**
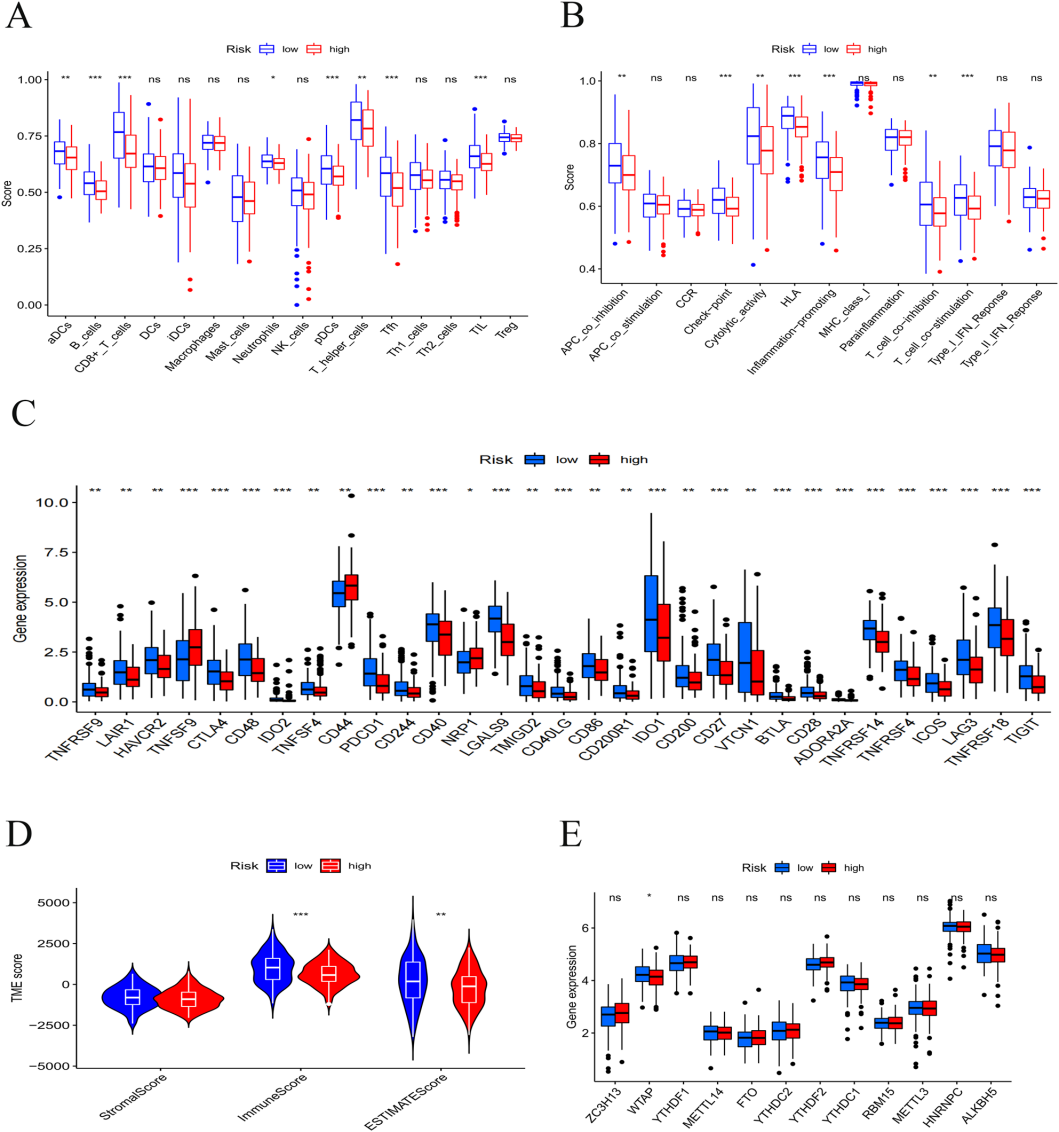
Boxplots of scores of immune cells (**A**) and immune-associated functions (**B**) in risk subgroups. (**C**) Expression of immune checkpoints among two risk subgroups in CESC patients. Associations between risk signature immune microenvironment (**D**) and m6A-related genes (**E**).

ssGSEA was employed to calculate diverse immune cell subpopulations, cellular functions, pathways and other factors to deeply figure out the relationship between prognostic risk score model and immunological state. As shown in Fig. 7A, the scores of most immune cells were largely different between the two risk subgroups. Not only the proportion of immune cell subpopulations, but also the level of immune-related functions in the high-risk subgroup was mostly lower than those in the low-risk subgroup (Fig. 7B). For immune checkpoints, all identified immune-related genes were expressed at a lower level in the high-risk subgroup, except TNFSF9, CD44 and NRP1 (Fig. 7C).

Both immune micro-environments including stromal and immune scores and M6A-related genes played a critical role in regulating the proliferation and invasion of tumor cell. Through the existing cell infiltration data analysis, the ESTIMATE score model showed that the TME score of low-risk was higher than that in the high-risk group (Fig. 7D). For expressed levels of the M6A-related gene, WTAP were lower in the high-risk subgroup than those in the low-risk subgroup (Fig. 7E).

### Tumor mutation burden (TMB) with survival outcomes

**Figure 8:**
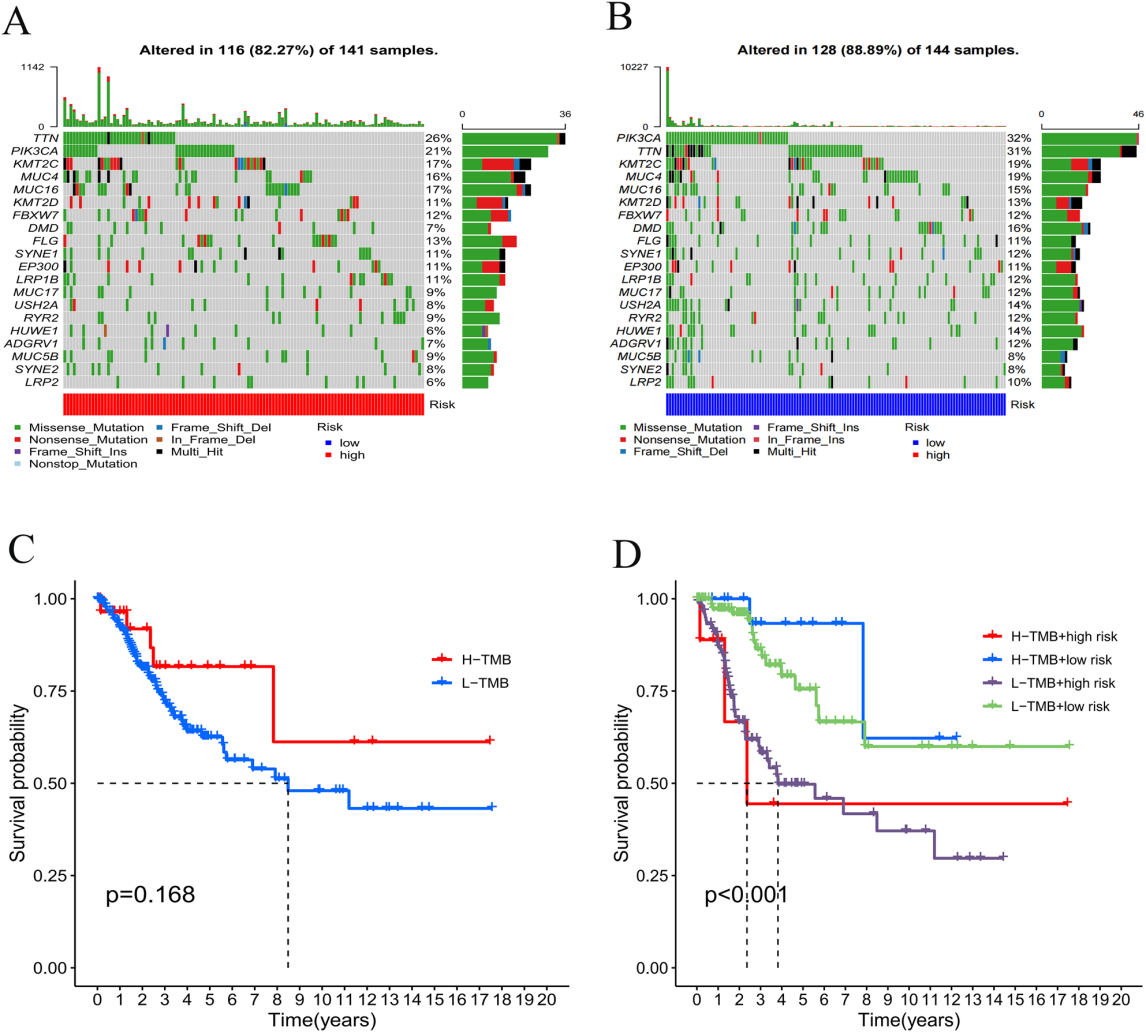
Correlation of tumor mutation burden (TMB) with survival outcomes. (**A**) Waterfall chart of somatic mutations of necroptosis-related genes. The bar plot above showed the tumor mutation burden. The right number represented mutation frequency individually. (**C**) High TMB group was associated with higher survival probability. (**D**) The H-TMB + low risk group had the highest 2-year survival probability trend of the four subgroups.

The maftools package was employed within R package maftools to display the mutation data findings. An exhaustive analysis on the gene mutation data of the CESC samples showed that the top 20 mutated genes were TTN, PIK3CA, KMT2C, MUC4, MUC16, KMT2D, FBXW7, DMD, FLG, SYNE1, EP300, LRP1B, MUC17, USH2A, RYR2, HUWE1, ADGRV1, MUC5B, SYNE2, LRP2. The results showed that the low-risk subgroup had a greater mutation frequency in the top 20 mutated genes than that in the high-risk group. (Fig. 8A,B). An examination of the Kaplan–Meier survival curves indicated a substantial difference between the two TMB subgroups (Fig. 8C), and the H-TMB + low risk group had the highest 2-year survival possibility trend in the four subgroups (Fig. 8D).

## Discussion

Cervical cancer is one of the leading causes of cancer deaths among women, and currently it is the fourth most common malignant tumor for torment women all over the world, with 604, 000 new diagnosed cases and approximately 342, 000 deaths reported in 2020 only^23^. CESC is considered the most common subtype of cervical cancer. The overall survival of CESC tends to be pessimistic due to the absence of early reliable and prognostic indicators, but some development has been made. The representatives are the screening technology for cervical cancer and prophylactic vaccination of HPV vaccine.

Necroptosis has advantages and disadvantages, and its specific effect depends on the cancer type and progression stage. Numerous studies showed that the downregulation of key necroptotic members such as CYLD^24^, RIPK3^25^ and MLKL^26^ was common for some types of cancer, which might be one of the significant molecular mechanisms for tumor cells to escape necroptosis. Tumor necroptosis takes place in vivo and induces the immunosuppressive tumor microenvironment, playing a tumor-promoting role^27^. lncRNA is a key regulator in various biological processes of cells^28^. As an important regulatory molecule in tissues and cells of cervical cancer, lncRNA may become a molecular marker and therapeutic target for the diagnosis and prognosis of cervical cancer. Previous researches have widely developed LncRNAs differentially expression-based markers to predicte the prognosis of cervical cancer patients. A one-lncRNA (AC073320.1) signature could be used to predict lymph node metastasis (LNM) status and prognosis^29^. Another seven-lncRNA (HCG11, CASC15, HULC, LINC00173, LINC00189, LINC00905 and MIR22HG) signature has been proved to play a basic role in predicting the recurrence of CC and could be a potential prognostic biomarker^30^. Nevertheless, it remains unclear for what the role and the mechanism of necroptosis-related lncRNAs is in CC prognostic model.

This study screened out NRlncRNAs in CESC, and comprehensively analyzed the expression of NRlncRNAs in CESC and their relevance to a patient's prognosis, and successfully developed a unique prognostic risk model combining 11 NRlncRNAs(MIR100HG, LINC00996, LINC02688, SNHG30, HCG15, TUBA3FP, MIAT, DBH−AS1, ERICH6−AS1, SCAT1, LINC01702). Based on this prediction risk score model, the CESC patients were categorized into the high-risk and low-risk groups. After analyzing the differentially-expressed NRGs between the two risk subgroups, this study found that the prognostic model showed high accuracy in predicting the overall survival of CESC. Through gene set and functional enrichment analysis with the NRlncRNAs, immune-related BP and signaling pathways were observed to be dramatically different in the two risk subgroups. The mRNA-lncRNA co-expression network was built to investigate the potential relationship of the 11 NRlncRNAs in CESC. Finally, the levels of different immune cell infiltrations in CESC were investigated, at the same time, the immune functions detected, potential immune checkpoints detected and the expression level of M6A-related genes examined. It was the first NRlncRNA prognosis and immune response model for CESC patients, which may provide customized therapeutic strategies in treating CESC and offering clinicians the prognostic information earlier to improve clinical treatment effects. Based on the GSEA, it was found that the high-risk group exhibited significantly activated “adherence junction”, “focal adhesion”, and “TGF beta signaling pathway”. HPV induces changes in the expression levels of cell adhesion markers^31^, modulates composition and structure of keratinocyte junctions^32^ and perturbs the strength of cell–cell^33^, thus facilitating HIV embedded in cervical mucosa tissue^34^ and increasing the risk of cervical cancer. Focal adhesion kinase (FAK), a non-receptor cytoplasmic tyrosine kinase, plays a crucial role in cell adhesion, cell spreading and migration. Some previous studies proved that FAK overexpressed in different cancers and promoted the process of cancer invasion, migration and metastasis^35–37^, producing a low survival rate of cancer patients^38,39^. Meanwhile, it has been found that the significant over-expression of FAK in CESC cells was probably correlated to the carcinogenesis and the promotion of tumor metastasis, being a potential target for the treatment using anti-cancer drugs to inhibit the proliferation, migration and survival of cancer cell by inducing apoptosis^40^. In the early stage of tumor formation, TGF-β exerts a tumor suppressor effect in a large extension. In the late stage, however, it can change the extracellular matrix of tumor tissues, promote tumor invasion and metastasis by promoting neovascularization and weaken the killing effect of natural killer cells on tumor cells, and it can promote tumor grow by producing long-term immunosuppression to the host too^41,42^. TGF-β has a bidirectional regulator in different stages of cervical cancer. It is low-expressed in cervical tissues of patients with cervical intraepithelial neoplasia grade I–III although high-expressed in cervical cancer tissues^43^. In addition, TGF-β may be involved in the carcinogenesis and development of HPV infection-related cervical cancer by the interaction with the key pathogenic proteins (HPV16 E6, E7) in HPV infection^44,45^.

Additionally, it was also found that the risk signature was very rich in several immunoregulatory pathways such as antigen processing and presenting, chemokine signaling pathway and T cell receptor signaling pathway against cancers I. It can be reasonably hypothesized that NRlncRNAs signature was closely correlated with anti-tumor immunity in CESC.

In the context of necroptosis, a previous study discovered that specific molecules could be emitted to induce the adaptive immune response and stimulate antigen presenting cell (APC) to phagocytize tumor cell, and that signals released by necroptosis cells could be significant for surveillance against tumors^46–48^. Intra-tumoral necroptosis not only promoted tumor antigen loading through tumor antigen presenting cells based on RIPK1/ RIPK3 ectopic activation, but also enhanced DC-, and CD8 + T cell-mediated anti-tumor immunity function, which might provide a new idea for current T-cell-based immunotherapy^49–51^. These studies indicated that necroptosis was closely correlated with the anti-tumor immunity effect, implying that our hypothesis can be supported strongly.

ssGSEA was performed to further investigate the underlying relationship between necroptosis and immune cell infiltration in CESC. Nearly all infiltrated immune cells and immune-related functions of the high-risk group showed a considerable reduction in CESC tissues including B cells, CD8 + T cells, neutrophils pDCs and NK cells when compared with those in the low-risk group. It has been previously proved that the interaction of tumor-infiltrating immune cells with the microenvironment was effective in predicting malignant progression^52^ and that infiltrating lymphocytes were prognostic biomarkers^53^. Therefore, it is reasonable to conclude that the poor prognosis of CESC patients in the high-risk subgroup relates to the decrease in anti-tumor immunity degree.

In the early stage of cervical cancer, tumor can induce immune response. When human immunity is low, tumor can develop via immune escape^54^. However, there is still lack of effective treatments for patients diagnosed with advanced and recurrent cervical cancer, which may make an opportunity for the substitutional treatments including immune checkpoint inhibitor therapy. Programmed cell death 1 (PD-1) is a critically negative immunomodulatory factor, and its ligands consists of PD-L1 and PD-L2^55^. PD-L1 expression can be detected in most cervical squamous cell carcinomas^56^. PD-L1 on the surface of tumor cells attenuates T cell-mediated cytotoxicity binding to PD-1 on the surface of activated T lymphocytes, which forms an immunosuppressive tumor microenvironment and promotes tumor cell proliferation and immune escape^57^. A completed phase Ib clinical trial (Keynote-028) demonstrated that the general response rate of the treatment with anti-PD-1 therapy (pembrolizumab) was 14.3% and that 91% of patients with the recurrent or metastatic cervical cancer had a response time of more than 6 months^58^. The positive expression of PD-L1 and PD-1 antibodies were observed to produce clinical outcomes and prognostic significance in cervical cancer^59^. Expression levels of nearly all immune checkpoint molecules were largely lower in high-risk group than those in the low-risk group, implying that the prognostic NRlncRNAs model could predict the expression of immune checkpoints in CESC, which might be beneficial to the efficacy of immune checkpoint blockade therapy in patients with CESC and offer a better option for anti-tumor immunity and immunotherapy. However, experimental and clinical data needs to be more for further validating the role of the necroptosis-related prognostic lncRNAs signature in predicting immunotherapy response from CESC patients.

The tumor microenvironment (TME) refers to a specific biological environment formed by tumor cells in conjunction with infiltrating immune cells, stromal cells, blood vessels, extracellular matrix and secretory factors in the tumor occurrence and development^60,61^. Immune cells of TME was observed to play an important role in tumor growth, invasion, and the stromal component is associated with tumor angiogenesis and extracellular matrix remodeling, thereby promoting tumor metastasis^62,63^. The results revealed that patients in the two risk subgroups had a higher immune score, which indicated a better response to immunotherapy. Therefore, TME might be used to predict the prognosis and immunotherapy efficacy in CESC patients. Wang et al.^64^ found that m6A levels in cervical cancer tissues were associated with tumor stage, size, differentiation, lymphatic infiltration and recurrence. NRlncRNAs signature of the study can not only effectively predict the expression levels of the m6A-related genes ZC3H13, FTO, YTHDF2, RBM15, WTAP, YTHDF1, METTL14, YTHDC2, YTHDC1, and ALKBH5 in CESC, but provide the academia with new idea and method to treat CESC.

TMB is a valuable biomarker to predict the clinical efficacy of immune checkpoint blockade (ICB) therapy for patients with many solid cancers^65–69^. Clinical trials and preclinical studies have shown that patients with tumors of higher somatic TMB (e. g. melanoma, breast cancer, non-small cell lung cancer) had longer life or better progression-free survival (PFS)^70^. This study investigated TMB of two risk subgroups. It was found that the lower group had a higher TMB and that there was a higher possibility for patients with high TMB to survive based on the findings of survival analysis. These results validated that TMB could be used to screen CESC patients for immunotherapy and predict the effect of immunotherapy and the prognostic status. Overall, TMB-based immunotherapy may be a promising oncology treatment for CESC patients.

Despite that there is a strong prediction value in the established risk model, there are some shortcomings in the current study. The significant shortcomings in this study lie in the lack of experimental validation of the identified targets. The mechanism by which necroptosis regulates the exact process of CESC remains unclear yet. It needs to be elucidated by further experimental studies.

## Conclusions

In conclusion, this study constructs a novel necroptosis-related prognostic risk signature of eleven-lncRNAs which may lead to the accurate prediction of the prognosis and the immune response of CESC patients. It may be the first necroptosis-related lncRNA prognostic model for CESC. The findings may provide an insight into the specific role necroptosis-related lncRNAs plays in CESC, therefore promoting the prognosis improvement of CESC patients and optimizing the customized treatment regimens.

## Supplementary Information

Supplementary Information.

## Data Availability

The data for this study come from The Cancer Genome Atlas (TCGA) database (https://portal.gdc.cancer.gov/). All the data in this paper support the results of this study. Details of R software: R is a free software environment for statistical computing and graphics. It compiles and runs on a wide variety of UNIX platforms, Windows and MacOS. R version 4.1.2; link: https://www.r-project.org/.
